# A perplexing silence: unlocking student communication through teacher humor in the EFL classroom

**DOI:** 10.3389/fpsyg.2025.1721941

**Published:** 2026-01-12

**Authors:** Tianli Zhou, Shiyue Chen, Jing Cao

**Affiliations:** 1 School of Foreign Languages, Zunyi Medical University, Guizhou, China; 2 School of Humanities and Foreign Languages, Zhejiang Shuren University, Hangzhou, China; 3 School of Foreign Languages, Lanzhou Jiaotong University, Gansu, China

**Keywords:** affect-behavior gap, English as a foreign language, learning enjoyment, teacher humor, willing to communicate

## Abstract

A persistent paradox haunts English as a foreign language classrooms: students report high learning enjoyment yet exhibit a perplexing silence and low willingness to communicate. Grounded in Control-Value Theory, this study conceptualizes teacher humor as an external antecedent that shapes learners’ control-value appraisals, thereby enhancing enjoyment and indirectly fostering willingness to communicate (WTC). This study proposes and empirically tests a psychological mechanism that contributes to understanding this “affect-behavior” gap, positioning teacher humor as a significant pedagogical factor. Path analysis of 483 undergraduates confirmed a significant indirect effect: teacher humor (TH) significantly boosted English learning enjoyment (ELE), which in turn was a powerful predictor of willingness to communicate. The partial mediation model reveals that the association between teacher humor and communicative intent proceeds primarily through this affective pathway, with ELE functioning as a crucial mechanism that mediates the relationship between them. This research offers two critical contributions: 1) It provides a tangible, teacher-driven strategy to mitigate the prevalent issue of student reticence. 2) It illuminates the specific psychological pathway for affect-to-behavior conversion in a second language context, offering a valuable theoretical model for future research on classroom emotional dynamics.

## 1 Introduction

For years, a persistent paradox has haunted English as a foreign language (EFL) classrooms in China: teachers deliver energetic lectures, yet students remain passive observers, creating an atmosphere of silent disengagement (Peng and Woodrow, 2010). This predicament has become even more puzzling with the “affective turn” in second language acquisition, which has rightly highlighted the importance of positive emotions. Research confirms that students can and do experience high levels of English learning enjoyment (ELE) (Jiang and Li, 2017). This creates a critical scholarly and pedagogical puzzle: Why does positive classroom affect not translate into active communicative behavior? This study argues that a crucial “affect-behavior” gap exists, and the key to bridging it lies within the teacher’s agency.

The stakes of solving this puzzle are particularly high in China, the country with the world’s largest population of English learners (Zhu and Wang, 2019). While communicative language teaching is increasingly advocated, Chinese students are often stereotyped as reticent, a trait previously attributed to factors like language anxiety or cultural values such as face-saving (Liu and Jackson, 2008; Wen and Cl’ement, 2003). While insightful, these explanations often overlook the immediate, dynamic classroom environment. They fail to fully explain why even students who genuinely enjoy their classes hesitate to speak, suggesting that a crucial catalyst is missing to bridge the gap between passive enjoyment into active willingness to communicate (WTC) (Bieg et al., 2017).

This study proposes that this catalyst is a specific, yet under-researched, teacher behavior: teacher humor (TH). To date, research on teacher emotions and student emotions has run on parallel tracks, with limited focus on their direct interaction (Li, 2021a). While studies have shown that humor can enhance student enjoyment (Bieg et al., 2022), its role as a potential mechanism for unlocking communication in the Chinese EFL context remains unverified. It is unclear whether humor directly prompts students to speak, or if it works through a more nuanced affective pathway.

Therefore, this study moves beyond simply correlating variables. We propose and empirically test a specific psychological mechanism that contributes to understanding the “enjoyment-silence” paradox. We hypothesize that ELE acts as a critical mediator in the relationship between TH and WTC. Specifically, we argue that teacher humor’s primary power lies not in directly triggering communication, but in significantly amplifying students’ learning enjoyment, which in turn dismantles the affective barriers to speaking. By examining this pathway, this research aims to provide a tangible pedagogical strategy for teachers to break the classroom silence and offers a novel theoretical contribution to understanding the process of affect-to-behavior conversion in second language learning. Thus, the research questions are threefold:

*RQ 1*: What are the current levels of English learning enjoyment and willingness to communicate among Chinese university students, and is there a significant positive relationship between them?

*RQ 2*: Does teacher humor significantly predict students’ English learning enjoyment?

*RQ 3*: Does English learning enjoyment mediate the relationship between teacher humor and students’ willingness to communicate?

### 1.1 Theoretical framework

The Control-Value Theory (CVT), a prominent framework in educational psychology, is widely utilized to analyze the structure, antecedents, and outcomes of academic emotions (Pekrun, 2006; Pekrun and Raymond, 2014). This theory proposed that academic emotions are emotions evoked by learning processes or outcomes and are characterized by three dimensions: goal relevance, valence, and activation. Goal relevance differentiates emotions into process-oriented (e.g., boredom, enjoyment) and outcome-oriented (e.g., anxiety, sadness) categories. Valence distinguishes between positive (e.g., enjoyment) and negative (e.g., boredom) emotions, while activation classifies emotions as high-arousal (e.g., enjoyment, anxiety) or low-arousal (e.g., boredom, frustration). Within this framework, foreign language learning enjoyment (FLLE) is conceptualized as a positive, high-arousal, process-oriented emotion, highlighting its role as a dynamic affective state linked to learning experiences rather than outcomes (Li et al., 2021). This theoretical lens provides a structured approach to understanding how emotional dimensions are related and associated with language learning behaviors.

The CVT also elucidates the relationship between academic emotions and academic achievement. Regarding factors influencing learning emotions, CVT identifies two fundamental determinants: subjective control over achievement activities and their outcomes, and the subjective value attributed to these activities and outcomes. Subjective control refers to the perceived contribution to actions and outcomes, such as evaluations of one’s ability to master learning content (Skinner, 1996), and encompasses expectations and attributions related to causal relationships between achievement contexts, the self, and personal outcomes. Subjective value, in turn, denotes the perceived worth of actions and outcomes, exemplified by evaluations of task importance (Pekrun, 2006). From the perspective of appraisal-emotion linkages, CVT posits that control and value appraisals serve as prerequisites for emotion generation, while emotions are reciprocally associated with appraisal processes. In other words, a bidirectional, reciprocal relationship exists between appraisals and achievement emotions. Regarding the regulation of academic emotions, CVT classifies control and value appraisals as cognitive appraisals, which are inherently malleable. Consequently, learning emotions are amenable to regulation. Regulatory strategies include employing relaxation techniques or pharmacological interventions to attenuate emotions, restructuring expectations and attributions, mastering learning skills, and reducing task difficulty. Collectively, these aspects demonstrate that CVT not only emphasizes the bidirectional associations of emotions with individuals’ cognitive, psychological, motivational, and social resources but also addresses the ‘antecedents’ of emotions and their reciprocal relationships with their antecedents and consequences (Dewaele and Li, 2022).

To articulate the theoretical path in the current study, we integrate the construct of Teacher Humor into the CVT framework. TH, as a positive and engaging pedagogical strategy, can significantly influence the antecedents of academic emotions: control and value appraisals.

Firstly, TH can enhance subjective control. TH often involves reducing classroom tension, managing anxiety, and making complex material more accessible (Frymier et al., 2008). This can lead learners to perceive the learning task as less daunting and more manageable, thereby increasing their subjective control over the learning process and outcomes.

Secondly, it can boost subjective value. Humorous instruction is intrinsically enjoyable and memorable, which can heighten students’ interest and engagement, contributing to a greater perceived subjective value of the learning activity itself (Keller, 2010). Furthermore, a humorous teacher can cultivate a positive classroom environment, increasing the perceived utility value of participation and communication.

According to CVT, these elevated control and value appraisals serve as powerful prerequisites for experiencing positive emotions. Specifically, high control and high value appraisals are posited to be the strongest predictors of enjoyment (Pekrun, 2006). Therefore, by positively impacting both control and value appraisals, TH is theoretically linked to the generation of English learning enjoyment. Finally, as a positive, high-arousal emotion, ELE is known to foster approach-oriented behaviors (Pekrun and Raymond, 2014), which logically facilitates students’ Willingness to Communicate in the classroom.

Based on the Control-Value Theory—and the hypothesized mechanism that TH enhances students’ control and value appraisals, leading to increased enjoyment (as detailed above)—this study conceptualizes TH as an environmental antecedent that directly shapes ELE, ELE as the core positive emotion, and students’ WTC as the resulting behavioral consequence. Hence, we propose the following specific hypotheses focusing on the direct observable links:

*Hypothesis 1*: Students’ ELE will significantly and positively predict their WTC.

*Hypothesis 2*: TH will significantly and positively predict students’ ELE.

*Hypothesis 3*: Students’ ELE will mediate the relationship between TH and WTC.

*Hypothesis 4*: A significant direct effect of TH on WTC will remain, even after accounting for the mediating role of ELE.

**Figure 1:**
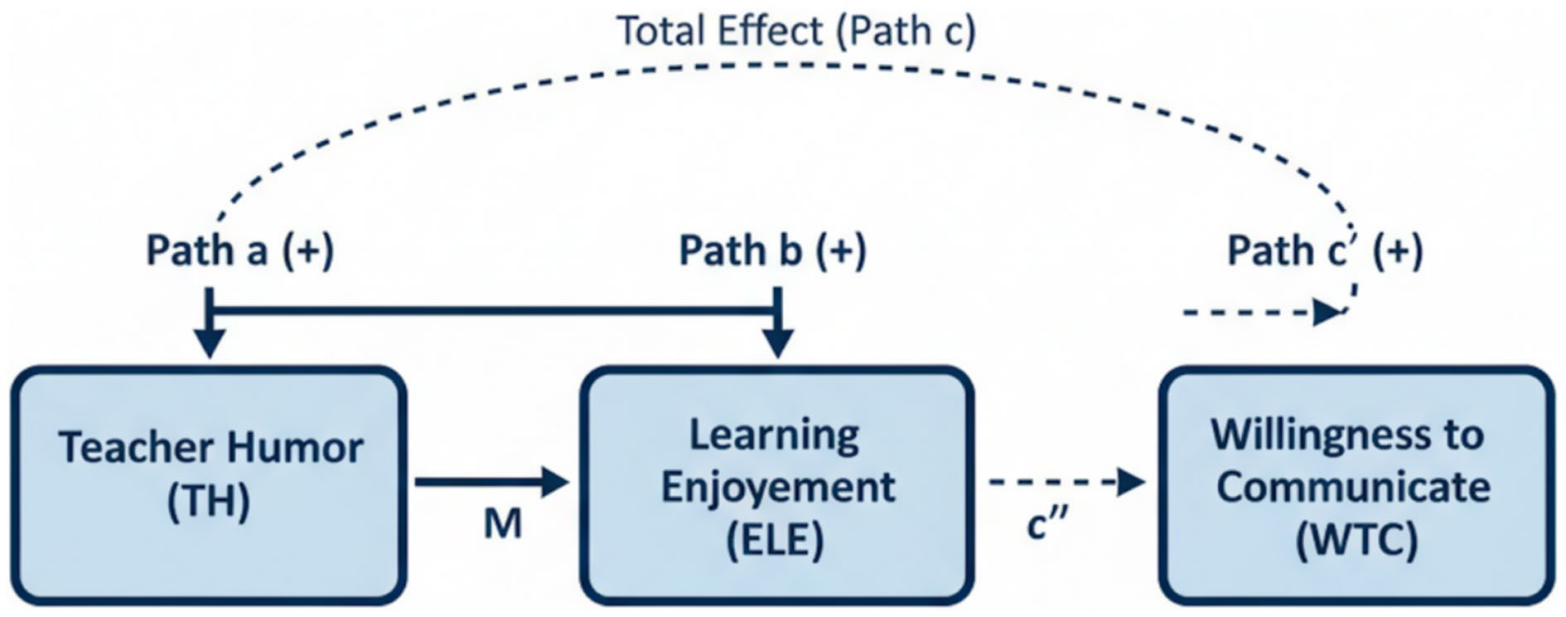
Hypothesized mediation model. X = teacher humor (TH), M = learning enjoyment (ELE). Path a: TH > ELE = WTC (+), Path c: ELE = TH = WTC (+). Indirect effect: TH = ELE = WTC (a * b). Total effect: C = c’ + (a * b). Predicted relationship direction: (+).

Figure 1 presents the hypothesized mediation model of this study.

## 2 Literature review

### 2.1 Studies on EFL students’ English leaning enjoyment and willingness to communicate

The study of foreign language enjoyment, rooted in the field of positive psychology, originated with Csikszentmihalyi’s (2014) seminal work defining “enjoyment” as a positive psychological state arising from the satisfaction of physiological or environmental needs. Dewaele and MacIntyre (2014) pioneered its application to Second Language Acquisition (SLA), conceptualizing FLE as an “emotional key” to unlocking learning potential by fostering a safe and enjoyable learning environment that encourages linguistic exploration. Subsequent theoretical developments have deepened its conceptual framework: Dewaele and MacIntyre (2016) emphasized FLE as reflecting learners’ intrinsic motivation to overcome challenges through problem-solving, while Jin and Zhang (2018) refined it as a context-specific emotional experience uniquely tied to foreign language learning tasks. Currently, FLE is widely recognized as the most extensively investigated positive emotion in SLA research (Dewaele and Li, 2020). Under China context, Li and Han (2022a) demonstrated comparable FLE levels (*M* = 3.59) between online and traditional classroom learners, with FLE positively predicting English proficiency. Notably, Li (2022) observed that while Chinese learners exhibit moderate FLE levels, these are lower than international averages, suggesting potential influences of educational contexts on emotional expression.

The concept of willingness to communicate originated in first language (L1) communication research, where it was defined as an individual’s stable personality trait for interaction (McCroskey and Richmond, 1987). In second language (L2) contexts, MacIntyre et al. (1998) proposed its dual “state–trait” characteristics: as a trait, it reflects learners’ stable communicative tendencies; as a state, it depends on dynamic contextual readiness. Building on this, L2-WTC was conceptualized as “the readiness to engage in communication with a specific interlocutor in an L2 at a particular time” (MacIntyre et al., 1998, p. 547). To explain this complexity, MacIntyre et al. (1998) developed the L2-WTC pyramid model, integrating linguistic proficiency, psychological variables, and socio-environmental factors. This model emphasizes that L2-WTC is shaped by both internal factors and external factors. Subsequent studies have validated the model’s comprehensiveness and dynamism, revealing L2-WTC as a critical mediating variable linking linguistic competence to actual communicative behavior. Despite the pyramid model’s theoretical comprehensiveness, empirical studies have predominantly focused on cognitive and affective variables at the individual level, with limited attention to how specific teacher characteristics and behaviors directly influence the situational readiness component of WTC.

In 2018, Dewaele and Dewaele (2018) first integrated FLE into the WTC predictive framework, revealing through a sample of London secondary school students (*N* = 189) that FLE was a strong predictor of WTC, second only to classroom climate (FLCA) and teachers’ frequency of L2 use. This study confirmed that enjoyment indirectly enhances communicative willingness by boosting learning motivation. Subsequently, Khajavy et al. (2017) demonstrated via multilevel analysis (*N* = 1,528) that classroom-level enjoyment significantly positively predicted WTC, while anxiety exerted negative effects only at the individual level, suggesting that positive emotions possess cross-contextual universality. Dewaele’s (2019) investigation in a Spanish context further revealed that FLE and teachers’ L2 use frequency jointly served as positive predictors of WTC, whereas classroom climate (FLCA) exhibited a negative association in this model, implying potential cultural variations in the role of emotional variables.

Recent research has substantially expanded understanding of the FLE-WTC relationship through more sophisticated methodological approaches and diverse cultural contexts. Zhang et al. (2024) employed structural equation modeling with 441 Chinese undergraduates, revealing that FLE positively predicted WTC through the mediating roles of communication confidence and motivation, thereby clarifying the psychological mechanism underlying this relationship. Lin and Wang (2025) further examined 690 Chinese EFL college students and found that FLE showed a stronger association with WTC than behavioral engagement, with both variables significantly predicting WTC. Their findings, grounded in Broaden-and-Build theory, suggest that enjoyment-focused strategies may enhance students’ communicative competence. Cross-cultural evidence from Bensalem et al. (2025) revealed that while both grit and FLE significantly predicted L2 WTC among Saudi (*n* = 228) and Moroccan (*n* = 218) students, FLE emerged as a stronger predictor for Saudi students, highlighting contextual influences on the emotion-communication nexus. Most notably, Li et al. (2025) conducted a large-scale study with 2,268 Chinese university students, demonstrating that enjoyment had the largest mediating effect in the relationship between classroom environment and WTC, followed by anxiety and boredom. Collectively, these studies established a dynamic interplay framework linking “enjoyment-motivation-communication” while revealing cultural and contextual variations in this relationship.

Additionally, Chinese studies highlight the uniqueness of teacher factors in the association between ELE and WTC. Liao’s (2023) research on Kunming high school students revealed a significant positive correlation between ELE and WTC, with teacher-related factors (e.g., pedagogical support) exerting significantly more substantial influences on FLE than self- or environmental factors. Liu’s (2022) study on junior high school students similarly identified teacher behavior as a prominent affective factor of FLE, echoing Dewaele and Dewaele (2018) findings in international samples. Additionally, Li’s (2022) study of 868 Chinese university students found teacher friendliness demonstrated a significant positive correlation with FLE and a significant negative correlation with FLLB, underscoring the decisive role of teacher–student interaction quality in shaping emotional experiences. Furthermore, some scholars proposed that teacher behaviors directly shape classroom emotional climates (Dewaele et al., 2022; Bieg et al., 2017, 2019). Culturally, Chinese learners exhibit greater reliance on teacher support (Liao, 2023).

Recent evidence further illuminates the bidirectional nature of teacher–student emotional dynamics. Solhi et al. (2024) employed an experience sampling method with nine English instructors and 162 students over 2 weeks, revealing a reciprocal relationship between foreign language teaching enjoyment (FLTE) and student FLE. Their dynamic structural equation modeling demonstrated that students’ high FLE levels correlated with higher L2 WTC, and notably, teachers with more development in FLTE had students with more development in their FLE and L2 WTC. This finding underscores that teacher emotions are not merely antecedents but are dynamically influenced by student emotional states and communicative behaviors, suggesting a complex feedback loop in the classroom emotional ecosystem. While teacher factors are recognized as influential, existing research has primarily examined general teacher behaviors (e.g., support, friendliness) rather than specific, differentiated teaching strategies such as distinct humor elements.

Based on the above literature review, this study identifies key limitations and research gaps in existing scholarship. Previous researchers have highlighted that L2 emotions and WTC are jointly associated with internal learner factors and external contextual factors. However, empirical studies examining the relationship of external factors with L2 emotions and WTC remain limited (Li, 2021b). What’s more, relevant studies remain scarce, failing to comprehensively capture the emotional states of interacting parties (Dewaele and Li, 2022). Even though recent studies have begun to address the reciprocal nature of teacher–student emotional dynamics (Solhi et al., 2024) and have identified classroom environment as a significant predictor of WTC through emotional mediation (Li et al., 2025). Yet, how specific, differentiated teacher behaviors systematically influence the FLE-WTC pathway remains unexamined. To address these gaps, this study aims to investigate, within the context of university English instruction, the relationships among teacher humor (as an external factor), FLE, and WTC among university students.

### 2.2 Studies on the relationship between teacher humor and EFL students’ performance

In recent years, research on the role of humor in EFL classrooms has gained increasing importance due to its potential to enhance language learning environments (Kabooha et al., 2024). By incorporating humor, teachers can foster a more relaxed classroom atmosphere, reduce student anxiety (Gunawardana, 2024), boost engagement, and strengthen teacher–student relationships. In addition, integrating humor into language pedagogy may lower learners’ affective filters, promoting greater motivation and knowledge retentionas noted by Heidari-Shahreza and Heydari (2018). Recent experimental evidence further demonstrates that humor discourse strategies effectively reduce students’ cognitive load and affective filtering, thereby improving learning efficiency and classroom engagement (Peng, 2025). Moreover, teacher care behavior represents another related dimension; fostering a supportive and humorous climate can alleviate learning anxiety and improve educational experiences (Wen et al., 2003).

Humor has also been demonstrated improvement the students and English teachers’ relationship significantly. Richmond et al. (2015) suggests that positive rapport between students and instructors correlates with perceptions of teaching effectiveness, implying that humor may contribute to building such relationships. Similarly, Tunnisa et al. (2019) emphasize that the appropriate use of humor helps establish rapport between teachers and students, thereby enhancing the creativity and effectiveness of the learning experience. This aligns with findings by Pranoto and Suprayogi (2020), Fata et al. (2018) and Purwanti et al. (2024) who indicate that humor can significantly improve classroom dynamics and facilitate smoother interpersonal relationships. These findings collectively demonstrate that humor serves not only as a pedagogical tool but also as a key element in cultivating supportive teacher–student relationships in EFL settings. While existing research establishes humor’s positive impact on rapport-building, most evidence remains descriptive and context-specific (Kabooha et al., 2024). The mechanisms through which different humor types of influence relationship quality across diverse cultural contexts warrant further quantitative investigation.

More recently international research has focused on the direct impact of teacher humor on FLE and WTC. Research has shown that humor correlates with positive teacher–student relations, which are, in turn, associated with student engagement and learning outcomes (Klassen et al., 2012). Fata et al. (2018) further highlight that students are more inclined to interact and express their feelings with teachers who employ humor, resulting in a more enjoyable learning experience. In a comparable vein, both Farahani and Abdollahi (2018) and Thuy (2022) provide evidence that the incorporation of humor significantly benefits EFL learners. Specifically, they find that humor not only enhances speaking abilities and willingness to communicate (Farahani and Abdollahi, 2018) but also fosters a positive classroom environment and increases learning engagement and active participation (Thuy and Thao, 2022). Dewaele et al.’s (2022) study at Kuwait University found that teachers’ predictable use of jokes and humor frequency positively correlated with FLE; conversely, a prolonged absence of teacher humor led to cumulative declines in FLE. Together, these studies underscore humor’s essential role in increasing enjoyment and engagement in language learning settings (Damanik et al., 2025), suggesting that effective use of humor by teachers promotes greater student disposition toward communication and participation. Despite accumulating evidence linking humor to FLE and WTC, studies have largely treated humor as a monolithic construct. The differential effects of specific humor strategies on distinct emotional and motivational outcomes remain underexplored, particularly through quantitative methods that can establish effect sizes and causal pathways.

Expanding on this line of inquiry, Neff and Dewaele (2023) examined which humor strategies prove most effective, finding that spontaneous humor, memes, and cartoons were most strongly endorsed by learners, with foreign language enjoyment and attitudes toward in-class humor exerting greater influence on strategy preferences than L2 proficiency levels. Therefore, the effectiveness of humor in EFL contexts extends to cultural and humor type considerations. The nuances of humor in varied cultural interactions further emphasize the importance of context when implementing humor as a teaching strategy (Peng, 2007; Moalla, 2014). Longitudinal evidence has begun to map the causal path from teacher humor to students’ achievement emotions. Bieg et al. (2017, 2019) showed that curriculum-relevant humor consistently raised enjoyment and dampened boredom or anger, whereas aggressive humor reversed these effects, establishing that the emotional impact depends on the humor style employed. Daumiller et al. (2020) extended this finding by demonstrating that the same content-aligned humor also lifts perceived instructional quality, suggesting that the emotional gains are not a mere side-effect but part of a broader improvement in the teaching–learning process. However, the positive trajectory is not automatic for every humorous cue, Bao et al. (2023) recently warned that only humor tightly integrated with course content produces measurable gains in engagement, while off-topic or self-disparaging jokes can nullify the benefits. Collectively, the literature frames curriculum relevance as a necessary boundary condition for humor to enhance both students’ emotions and instructional quality.

However, the literature consistently demonstrates that the effectiveness of humor depends on its style. Scholars commonly distinguish between prosocial/affiliative humor (which builds rapport and reduces anxiety) and instructional/content-relevant humor (which aids learning and enhances instructional quality). And although prior work has established that teacher humor fosters a positive classroom climate and strengthens teacher–student rapport in EFL contexts (Jiang, 2022; Reddington and Waring, 2015), the evidence remains qualitative and undifferentiated. How humor regulate two key language-learning emotions—students’ immediate enjoyment of classroom activities and their situated willingness to communicate (WTC)—has not been subjected to targeted, quantitative scrutiny. Recent studies have begun identifying effective humor strategies and their motivational impacts (Neff and Dewaele, 2023; Kabooha et al., 2024), yet the size and pathways of any causal effects linking specific humorous instruction styles to enjoyment-driven WTC remain unknown. Consequently, the size and pathways of any causal effects linking humorous instruction to enjoyment-driven WTC are still unknown. Hence, this study provides valuable insights into that area.

## 3 Methods

### 3.1 Participants and sampling strategy

The study employed a convenience sampling approach to recruit 483 Chinese college student volunteers from three undergraduate universities strategically chosen to represent regional diversity across China. While convenience sampling limits generalizability, it is a pragmatic and commonly accepted approach in preliminary scale development studies (Carpenter, 2018). The primary purpose of this initial validation is to establish basic psychometric properties and factor structure rather than population parameter estimation, which would require probability sampling methods.

The participants’ age ranged from 18 to 22 years old (*M* = 20.3, *SD* = 1.1), typical for Chinese undergraduate students. Students were recruited from a broad range of disciplines across the university (e.g., humanities, engineering, and business). Socio-economic background was not directly measured, but the selection of three universities across Eastern (first tier), Central (modal), and Western (developing) regions aimed to capture a spectrum of socio-educational contexts present in China. University A (Eastern region, *n* = 165): A comprehensive university located in a first-tier coastal city with relatively high English education resources and international exposure. University B (Central region, *n* = 162): A provincial university in a second-tier inland city representing the modal Chinese university context. University C (Western region, *n* = 156): A regional university in an economically developing area where English education resources are more limited.

The sample was distributed across grade levels as follows: freshmen (*n* = 95, 19.7%), sophomores (*n* = 114, 23.6%), juniors (*n* = 271, 56.1%), and seniors (*n* = 3, 0.6%). The predominance of junior students and near-absence of seniors because of the structural reality of college English education in China. Most Chinese universities concentrate required college English courses in the first four semesters (freshman and sophomore years), with some institutions extending into the junior year. By senior year, the vast majority of students have completed their college English requirements and are no longer enrolled in these courses, making recruitment from this population extremely challenging through classroom-based convenience sampling. The junior-year predominance (56.1%) may also own to the optimal timing for humor perception assessment: these students have accumulated sufficient experience with college English instruction (4–6 semesters) to form stable perceptions of teacher humor, while remaining actively engaged in English learning contexts.

As shown in Table 1, participants’ English proficiency levels, indicated by CET participation, were distributed as follows: 66 students (13.7%) had not yet taken either CET-4 or CET-6, 111 students (23.0%) reported CET-4 scores ≥425 (the passing threshold), 102 students (21.1%) scored between 425 and 425 on CET-4, 104 students (21.5%) achieved CET-6 scores <425, and 100 students (20.7%) achieved CET-6 scores ≥425. This distribution reflects the progressive nature of CET participation in Chinese higher education. CET-4 is typically attempted in the second semester of freshman year or later, explaining why 13.7% of our sample (primarily freshmen) had not yet taken the exam. The relatively balanced distribution across CET-4 and CET-6 achievers suggests our sample captures a range of English proficiency levels, from lower-intermediate (CET-4 passers) to advanced (high-scoring CET-6 achievers). This proficiency diversity is advantageous for scale validation, as it ensures the ETHPS is evaluated across the spectrum of English learners rather than only high- or low-proficiency students.

**Table 1:** Participants’ demographic information (*N* = 483).

Characteristics		Frequency	Percent	Valid percent	Cumulative percent
Gender	Male	176	36.4	36.4	36.4
	Female	291	60.2	60.2	96.7
	Others	16	3.3	3.3	100
Grade	Freshman	95	19.7	19.7	19.7
	Sophomore	114	23.6	23.6	43.3
	Junior	271	56.1	56.1	99.4
	Senior	3	0.6	0.6	100
CET level	None	66	13.7	13.7	13.7
	CET-4,<425	111	23	23	36.6
	CET-4, ≥425	102	21.1	21.1	57.8
	CET-6,<425	104	21.5	21.5	79.3
	CET-6, ≥425	100	20.7	20.7	100

### 3.2 Instruments

The measurement tool required for this study is a questionnaire scale, and thus three scales were employed in this research: the Student Foreign Language Enjoyment Scale, the Willingness to Communicate Scale, and the English Teachers’ Humor Perception Scale.

#### 3.2.1 Foreign Language Enjoyment Scale

To measure learners’ ELE states during English instruction, the present study employed the Chinese adaptation of the Foreign Language Enjoyment Scale (CFLES; Li et al., 2018). This instrument, derived from Dewaele and MacIntyre’s (2014) original FLES, underwent comprehensive validation across diverse Chinese EFL learning environments. The CFLES contains 11 items structured into three dimensions: personal enjoyment (FLE-Private), teacher-related enjoyment (FLE-Teacher), and classroom atmosphere enjoyment (FLE-Atmosphere). Participants indicate responses using a 5-point Likert system ranging from *strongly disagree*(1) to *strongly agree*(5). Initial psychometric evaluation by Li et al. (2018) with 1,718 senior high school students demonstrated robust psychometric properties, including satisfactory construct, discriminant, and convergent validity, alongside good internal consistency (*α* = 0.83). Subsequent validation studies confirmed its reliability across different educational stages: Wei et al. (2019) recorded *α* = 0.81 among 832 junior secondary students, while Hung’s (2020) investigation with 1,464 university students yielded *α* = 0.90. In the present study, Cronbach’s alpha coefficients demonstrated good reliability: overall FLE (*α* = 0.87), while the results of KMO is 0.85.

#### 3.2.2 Willing to Communicate Scale

The present study employed Peng and Woodrow’s (2010) Willingness to Communicate Scale to measure college students’ English communication willingness. The scale developed by Peng and Woodrow (2010) involves a large-scale investigation of willingness to communicate (WTC) in Chinese English-as-a-foreign-language (EFL) classrooms, making it suitable for the current research context. The WTCS (Peng and Woodrow, 2010) is a 5-point Likert scale comprising two dimensions: WTC1 (Willingness to Communicate in English during meaning-focused activities) and WTC2 (Willingness to Communicate in English during form-focused activities), with 10 items in total (*α* = 0.88). Reliability and validity analysis of the collected data in this study (*n* = 483) yielded *α* = 0.95 and KMO = 0.93, indicating excellent psychometric properties for the current investigation.

#### 3.2.3 English Teachers’ Humor Perception Scale

Through literature review and preliminary surveys, limited established scale targeting Chinese English learners was identified. Therefore, this study developed the English Teachers’ Humor Perception Scale (ETHPS), which captures Chinese EFL learners’ perceptions across behavioral and affective dimensions. The development of the ETHPS items followed a rigorous multi-stage process to ensure content validity, cultural appropriateness, and psychometric soundness. Initial items were generated based on comprehensive review of humor literature, including Frymier et al.’s (2008) Humor Behaviors Scale (HBS) and Edwards and Martin’s (2014) Humor Styles Questionnaire (HSQ). A preliminary pool of 25 items was created, covering both behavioral manifestations of teacher humor and students’ affective responses to humor in English language learning contexts.

A panel of three experts was convened to evaluate the initial item pool for content validity. The panel consisted of: One professor specializing in English language teaching methodology with 15 years of experience; One associate professor in applied linguistics with expertise in classroom interaction; One senior researcher in educational psychology focusing on affective factors in language learning. Experts independently reviewed each item using a structured evaluation form assessing: (a) clarity of wording, (b) relevance to teacher humor perception, (c) cultural appropriateness for Chinese EFL contexts, and (d) potential redundancy with other items. Items were rated on a 4-point scale (1 = not adequate, 4 = very adequate). Items receiving a mean rating below 3.0 or showing substantial inter-expert disagreement (*SD* > 1.0) were flagged for revision or deletion. Based on expert feedback, six items were eliminated due to cultural inappropriateness or ambiguity, and eight items were revised for clarity. For example, an initial item “The teacher’s jokes make me laugh out loud” was revised to “The teacher’s humor makes me feel cheerful in class” to better capture affective response rather than merely behavioral reaction, which experts noted might be culturally constrained in Chinese classroom settings.

Then, semi-structured interviews were conducted with five Chinese college English teachers (three females, two males; teaching experience: 3–12 years) to gather qualitative feedback on item comprehensibility, relevance, and face validity. Each interview lasted approximately 30–45 min and followed a structured protocol. Teachers were first asked to read all items and identify any ambiguous or confusing wording. They then rated each item’s relevance to their students’ experiences on a 5-point scale. Open-ended questions elicited specific feedback on cultural sensitivity and potential improvements. Interview data were analyzed using content analysis. Key findings indicated that: (a) Teachers emphasized the importance of distinguishing between humor’s cognitive benefits (e.g., memory enhancement, attention maintenance) and emotional benefits (e.g., anxiety reduction, rapport building). This feedback reinforced the theoretical rationale for a two-dimensional structure. (b) Three items were identified as potentially overlapping in meaning and were subsequently consolidated or reworded. (c) Teachers suggested incorporating items specifically addressing humor’s role in reducing English speaking anxiety, leading to the addition of item 7 (“英语教师幽默帮助我减轻用英语发言的焦虑/The English teacher’s humor helps reduces my anxiety about speaking English”).

Based on expert review and teacher interviews, the item pool was refined to 19 items. All items were originally developed in English and then translated into Chinese by two bilingual researchers. Back-translation was performed by an independent translator, and discrepancies were resolved through discussion to ensure semantic equivalence. Special attention was paid to culturally appropriate expressions of humor appreciation in Chinese educational contexts.

Prior to formal administration, a pilot study (*N* = 290) was conducted. The present study employed principal component analysis (PCA) with Varimax rotation to validate the factor structure of the ETHPS. Key findings are presented in Table 2. The total variance explained indicates that two principal components (eigenvalues >1) were extracted from the 19-item scale, accounting for a cumulative variance of 73.023% (pre-rotation cumulative: Component 1 = 67.193% + Component 2 = 5.830%). This outcome exceeds the 60% threshold commonly required for social science scales, demonstrating that the factor structure effectively captures the original data’s information. After rotation, the eigenvalue distribution was optimized: Component 1 (eigenvalue = 7.37) explained 38.77% of the variance, while Component 2 (eigenvalue = 6.51) accounted for 34.25%. The post-rotation variance contributions became more balanced, confirming that the Varimax rotation improved the factor structure’s interpretability.

**Table 2:** Total variance explained.

Component	Initial eigenvalues			Rotation sums of squared loadings		
	Total	% of variance	Cumulative %	Total	% of variance	Cumulative %
1 (pilot 1)	12.767	67.193	67.193	7.367	38.772	38.772
2 (pilot 1)	1.108	5.83	73.023	6.508	34.251	73.023
1 (pilot 2)	10.530	70.198	70.198	6.380	42.534	42.531
2 (pilot 2)	1.093	7.289	77.487	5.243	34.955	77.487

Extraction method: principal component analysis.

However, significant cross-loadings (>0.40) on behavioral and affective dimensions were observed for items 8, 10 and 13. Given their lack of discriminant validity (max load difference = 0.17), these items were excluded to achieve a theoretically coherent factor structure. Item 9 (教师的幽默帮助我更好地记住单词和句型/ *The teacher’s humor helps me remember words and sentence patterns better*) showed a minimal loading difference of 0.108 between factors, necessitating verification of whether it measures both behaviors and outcomes. During the expert review and pilot interview phases, this item received consistently high ratings for relevance (*M* = 4.67 out of 5 from teacher interviews) and was specifically highlighted by four out of five interviewed teachers as capturing a critical function of humor in their classrooms. Chinese EFL teachers particularly emphasized humor’s mnemonic value given the challenges students face in vocabulary acquisition, making this item contextually essential for the Chinese learner population.

In addition, item 9 captures a unique transitional aspect of teacher humor perception that bridges behavioral and affective dimensions. Memory enhancement through humor inherently involves both cognitive-behavioral processing (the actual mnemonic function) and affective engagement (the emotional resonance that facilitates encoding). This dual nature reflects the interconnected reality of humor’s effects in educational contexts rather than a psychometric flaw. Humor research suggests that memorable learning experiences often result from the interaction between cognitive and emotional processes (Schmidt, 1994; Wanzer et al., 2010), which this item authentically captures. Moreover, the study attempted to remove item 9 but observed a decrease in Cronbach’s Alpha (*α* = 0.96), prompting retention through semantic refinement instead (教师的幽默提升了我记忆单词和句型的能力/ *The teacher’s humor enhances my ability to remember words and sentence patterns*).

After these modifications, the ETHPS finally includes 15 items with two core dimensions emerged as Affective Interaction Efficacy and Humor Implementation Efficacy, forming a stable and valid two-factor structure (see Appendix 1). The two factors are conceptualized based on the theoretical distinction between humor’s social–emotional role and its pedagogical role in education:

Factor 1: Affective Interaction Efficacy: This factor captures the socio-emotional function of humor, reflecting its ability to build rapport and create a low-anxiety environment, consistent with Affiliative Humor in the broader literature.

Factor 2: Humor Implementation Efficacy: This factor captures the instructional function of humor, reflecting the teacher’s ability to integrate humor effectively with course content to enhance learning and engagement, consistent with Curriculum-Relevant Humor (Bieg et al., 2017; Daumiller et al., 2020).

Reliability and validity analysis of the pilot data revealed excellent internal consistency (*α* = 0.97) and a KMO value of 0.956 (>0.9, indicating exceptional suitability for factor analysis), demonstrating strong partial correlations among variables. In addition, its cumulative variance explained = 77.49% (Table 2).

##### 3.2.3.1 Structural validity: confirmatory factor analysis (CFA)

To validate the two-factor structure identified by the Exploratory Factor Analysis (EFA), a Confirmatory Factor Analysis (CFA) was conducted on a separate sub-sample using AMOS (Version 26). The hypothesized two-factor model (F1: Affective Interaction Efficacy and F2: Humor Implementation Efficacy) was tested.

The model fit indices indicated that the hypothesized two-factor structure provided an acceptable fit to the data: *χ* ^2^ (81) = 816.154, *p* < 0.001. Although the CMIN/DF ratio was high at 9.170 (a common issue with large sample sizes), the incremental fit indices reached acceptable thresholds: CFI = 0.907 and IFI = 0.907 (≥0.90). The absolute fit index RMR was excellent at 0.023 (≤0.08). These results collectively support the structural validity of the two-factor model.

Convergent validity and internal consistency were assessed using standardized factor loadings (*λ*), Construct Reliability (CR), and Average Variance Extracted (AVE). All standardized factor loadings for the 15 items were statistically significant (*p* < 0.001), ranging from 0.806 to 0.887. As shown in Table 3, both factors demonstrated excellent reliability and convergent validity, with CR values above the 0.70 threshold and AVE values above the 0.50 threshold. For Factor 1, CR = 0.964 and AVE = 0.751; for Factor 2, CR = 0.938 and AVE = 0.717.

**Table 3:** Convergent validity and reliability of the ETHPS.

Factor	Items	Standardized factor loadings (*λ* range)	Construct reliability (CR)	Average variance extracted (AVE)
F1: Affective interaction efficacy	9	0.819–0.885	0.964	0.751
F2: Humor implementation efficacy	6	0.806–0.887	0.938	0.717
Threshold	–	≥ 0.70	≥ 0.70	≥ 0.50

CR, construct reliability; AVE, average variance extracted. Standardized factor loadings were all statistically significant (*p* < 0.001).

Discriminant validity was assessed using the Fornell and Larcker criterion (Table 4), comparing the square root of AVE ( $\sqrt{\mathrm{AVE}}$ ) for each factor with the inter-factor correlation (*r*). The correlation between the two factors was 0.853 (*p* < 0.001). Factor 1 passed the criterion ( $\sqrt{\mathrm{AVE}}$ =0.867 > 0.853). Factor 2 slightly missed the threshold ( $\sqrt{\mathrm{AVE}}$ =0.847 < 0.853). Given the high overall model fit and the strong theoretical distinction between the two constructs, the CFA model was retained, indicating that while the factors are highly correlated, they remain conceptually distinct.

**Table 4:** Discriminant validity assessment (Fornell–Larcker criterion).

Factor	F1: affective interaction efficacy	F2: humor implementation efficacy
F1: Affective interaction efficacy	**0.867** ( $\sqrt{\mathrm{AVE}}$ )	-
F2: Humor implementation efficacy	0.853 (*r*)	**0.847** ( $\sqrt{\mathrm{AVE}}$ )

Values on the diagonal (in bold) represent the square root of the average variance extracted $\sqrt{\mathrm{AVE}}$ . Values below the diagonal are the inter-factor correlations (*r*).

### 3.3 Data collection and analysis

Researchers utilized a convenience and snowball sampling strategy to recruit university student volunteers across three universities. Recruitment was conducted through the investigator’s personal and professional networks, including direct students, colleagues, friends, and collaborators at these universities. To ensure a broad geographical representation, data collection was conducted online using the Wenjuanxing platform.^1^ All participants provided informed consent prior to starting the survey. They were informed of the voluntary nature of their participation and the confidentiality of their responses. The questionnaire comprised four sections: 1) Demographic Information (Gender, grade level, and English proficiency, i.e., CET level). 2) English learning enjoyment (11 items). 3) Willingness to Communicate (10 items). 4) Perception of Teacher Humor (15 items).

A total of 500 responses were initially collected online. To maintain data quality and rigorousness, the following inclusion and exclusion criteria were applied to identify and exclude “invalid” responses:

Inclusion criteria: Responses were only included if the participant confirmed they were a current university student learning English as a foreign language.

Exclusion criteria

1. Duplicate Responses: Submissions identified through matching IP addresses or identical response times were automatically blocked by the Wenjuanxing platform or manually identified and removed.
2. Incomplete Submissions: Responses with more than 5% missing values across the total 36 scale items were excluded.
3. Invalid Responses (Straight-lining or rushing): Submissions where all scale items shared the same response option (e.g., all 5 s) or submissions completed in less than 180 s (a predetermined minimum time based on pilot testing) were flagged as invalid and removed. After applying these screening criteria, 17 submissions were excluded (including 5 duplicates and 12 invalid/incomplete responses). A final sample of 483 valid questionnaires was retained for analysis. What’s more, given the convenience sampling method employed through professional networks, a traditional response rate (i.e., the percentage of those invited who completed the survey) cannot be accurately calculated. However, the data retention rate was 96.6% (483/500), indicating high engagement and successful collection among those who started the survey.

The analytical procedures employed a sequential quantitative approach to address the three research questions. Firstly, all multi-item constructs—learning enjoyment (11 items), communication willingness (10 items), and teacher humor (15 items)—underwent reliability assessment, demonstrating internal consistency (Cronbach’s *α* = 0.87, 0.95, and 0.97, respectively). The overall Cronbach’s *α* = 0.96. Composite scores were computed as arithmetic means to represent latent variables. Descriptive statistics and correlation analyses were subsequently performed on several variables. A linear regression analysis was then conducted to examine the predictive effect of ELE on WTC, and the predictive effect of TH on ELE. Finally, a mediation analysis was performed to examine the mediating role of ELE in the relationship between TH and WTC. All analyses were conducted in SPSS 27.0. Mediation analysis was performed using PROCESS macro (Model 4.2), rather than Structural Equation Modeling (SEM). PROCESS, being based on Ordinary Least Squares (OLS) regression, is ideally suited for testing a simple and directed mediation path. Given that this study utilized mean scores of multi-item scales as manifest variables, PROCESS, in conjunction with the Bootstrap method, provides a robust estimation of the indirect effect and its confidence intervals, offering a concise and sufficient framework for testing the research hypotheses.

### 3.4 Ethical considerations

This study adhered to strict ethical standards throughout its implementation. Prior to participation, all participants provided informed consent after being fully briefed on the study’s purpose and procedures. Their anonymity and the confidentiality of their data were rigorously protected using coded identifiers. Participation was entirely voluntary, with the right to withdraw at any time without penalty. The research protocol was approved by the Institutional Review Board of the researchers’ institution to ensure all procedures minimized risk and respected participant welfare.

## 4 Results

This section presents the descriptive statistics and inter-correlations among the key variables, followed by the tests of the hypothesized model.

Table 5 displays the descriptive statistics for TH, ELE, and WTC. The mean scores on a 5-point Likert scale indicated that students perceived their teachers as having relatively high humor (*M* = 4.04, *SD* = 0.61) and reported moderate-to-high levels of English learning enjoyment (*M* = 3.54, *SD* = 0.52). However, students’ WTC was at a moderate level (*M* = 3.08, *SD* = 0.82), with greater variability compared to the other two variables. This finding aligns with the “affect-behavior gap” observed in EFL contexts, where high enjoyment does not always translate into high communication.

**Table 5:** Descriptive statistics.

Variable	*N*	Minimum	Maximum	Mean	Std. Deviation
ELE	483	1.82	5	3.5391	0.52127
WTC	483	1	5	3.0847	0.82201
TH	483	2.4	5	4.0396	0.61144

Table 6 presents the Pearson correlation coefficients among the variables. Consistent with Hypothesis 1, ELE was significantly and positively correlated with WTC (*r* = 0.62, *p* < 0.001). Furthermore, TH showed a significant positive correlation with ELE (*r* = 0.56, *p* < 0.001), consistent with the direction hypothesized in Hypothesis 2. TH was also significantly and positively correlated with WTC (*r* = 0.47, *p* < 0.001). These preliminary correlations provide initial support for the relationships specified in the hypothesized model.

**Table 6:** Correlations among study variables.

Variable	ELE	WTC	TH
ELE	1		
WTC	0.623** [0.564, 0.680]	1	
TH	0.555** [0.482, 0.610]	0.470** [0.386, 0.528]	1

*N* = 483. Values in the first line are Pearson correlation coefficients (*r*). Values in parentheses are the 95% bias-corrected and accelerated (BCa) confidence intervals (LL, UL). ELE, English learning enjoyment, WTC, willingness to communicate, TH, teacher humor. Effect size benchmarks (Cohen, 1988): *r* = 0.10 is a small effect, *r* = 0.30 is a medium effect, and *r* = 0.50 or greater is a large effect. **p < 0.01 (two-tailed).

Given that all constructs were measured using self-report questionnaires administered in a single survey session, we assessed the potential threat of common method bias (CMB) using Harman’s single-factor test, the most widely employed statistical method for detecting common method variance in survey research (Podsakoff et al., 2003).

All items from the three scales (ETHPS: 15 items; ELE: 11 items; WTC: 10 items; total = 36 items) were entered into an exploratory factor analysis using principal components extraction with no rotation. The underlying logic of this test is that if substantial common method variance exists, a single general factor should emerge from the unrotated factor solution, accounting for the majority of covariance among variables. Podsakoff and Organ (1986) suggest that if the first factor accounts for more than 50% of total variance, common method bias may be a serious concern.

Results (Table 7) indicated that seven factors with eigenvalues greater than 1.0 emerged from the unrotated solution, collectively explaining 77.154% of total variance. Critically, the first unrotated factor accounted for 43.630% of variance, below the conservative 50% threshold. The second factor explained an additional 14.107% of variance, bringing the cumulative variance to 57.737% for the first two factors. The emergence of multiple substantial factors, rather than a single dominant factor, suggests that the covariance among measures is not primarily attributable to a common method effect. In summary, Harman’s single-factor test provides evidence that common method bias, while present, does not appear to be a severe threat to the validity of our findings.

**Table 7:** Harman’s single-factor test: total variance explained by unrotated principal components.

Initial Eigenvalues				
Component	Total	% of variance	Cumulative %	Interpretation
1	15.707	43.630	43.630	Below 50% threshold; CMB not dominant
2	5.078	14.107	57.737	Second factor explains substantial additional variance
3	2.025	5.625	63.362	
4	1.379	3.832	67.193	
5	1.366	3.794	70.987	
6	1.161	3.226	74.213	
7	1.059	2.941	77.154	Seven factors with eigenvalues >1.0
8–36	8.227	22.846	100.000	Remaining factors (eigenvalues <1.0)

*N* = 483. Total number of items = 36, comprising ETHPS (15 items), English learning enjoyment scale (11 items), and willingness to communicate scale (10 items).

### 4.1 Hypothesis 1: English learning enjoyment predicting willingness to communicate

To examine Hypothesis 1, which assumes that ELE significantly and positively predicts WTC, a simple linear regression analysis was conducted. The results, presented in Table 8, indicated that the model was statistically significant, *F*(1, 481) = 305.61, *p* < 0.001. ELE accounted for 38.9% of the variance in WTC (*R^2^* = 0.389). As hypothesized, ELE emerged as a highly significant positive predictor of WTC (*β* = 0.62, *t* = 17.48, *p* < 0.001). Specifically, for every one-unit increase in ELE, WTC was predicted to increase by 0.98 units. Therefore, Hypothesis 1 is fully supported by the data.

**Table 8:** Summary of simple linear regression analysis predicting willingness to communicate (WTC) from English learning enjoyment (ELE).

Variable	*B*	*SE B*	95% CI for *B*	*β*	*t*	*p*-value
(Constant)	−0.394	0.201	[−0.789, 0.001]	–	−1.959	0.051
ELE	0.983	0.056	[0.872, 1.093]	0.623	17.482	0.000***

*N* = 483. *R*^2^ = 0.389; adjusted *R*^2^ = 0.387; *F*(1, 481) = 305.614, ***p < 0.001. CI, confidence Interval; ELE, English learning enjoyment.

### 4.2 Hypothesis 2: teacher humor predicting English learning enjoyment

Hypothesis 2 hypotheses that TH would significantly and positively predict students’ ELE. A simple linear regression analysis was performed, and the results are summarized in Table 9. The model was found to be statistically significant, *F*(1, 481) = 206.01, *p* < 0.001. TH explained 30% of the variance in ELE (*R^2^* = 0.300).

**Table 9:** Summary of simple linear regression analysis predicting English learning enjoyment (ELE) from teacher humor (TH).

Variable	*B*	*SE B*	95% CI for B	*β*	*t*	*p*-value
(Constant)	1.610	0.136	[1.343, 1.877]	–	11.855	0.000***
TH	0.476	0.033	[0.411, 0.541]	0.548	14.353	0.000***

*N* = 483. *R*^2^ = 0.300; adjusted *R*^2^ = 0.298; *F*(1, 481) = 206.01, ***p < 0.001. CI, confidence interval; TH, teacher humor.

Consistent with the hypothesis, TH emerged as a highly significant positive predictor of ELE (*β* = 0.55, *t* = 14.35, *p* < 0.001). Specifically, for every one-unit increase in perceived TH, ELE was predicted to increase by 0.48 units. Therefore, Hypothesis 2 is fully supported by the data.

### 4.3 Hypotheses 3 and 4: mediating role of English learning enjoyment

To test Hypotheses 3 and 4 regarding the mediating role of ELE in the relationship between TH and WTC, a mediation analysis was conducted using PROCESS macro-Model 4 (Hayes, 2012) with 5,000 bootstrap samples to generate 95% bias-corrected confidence intervals.

**Figure 2:**
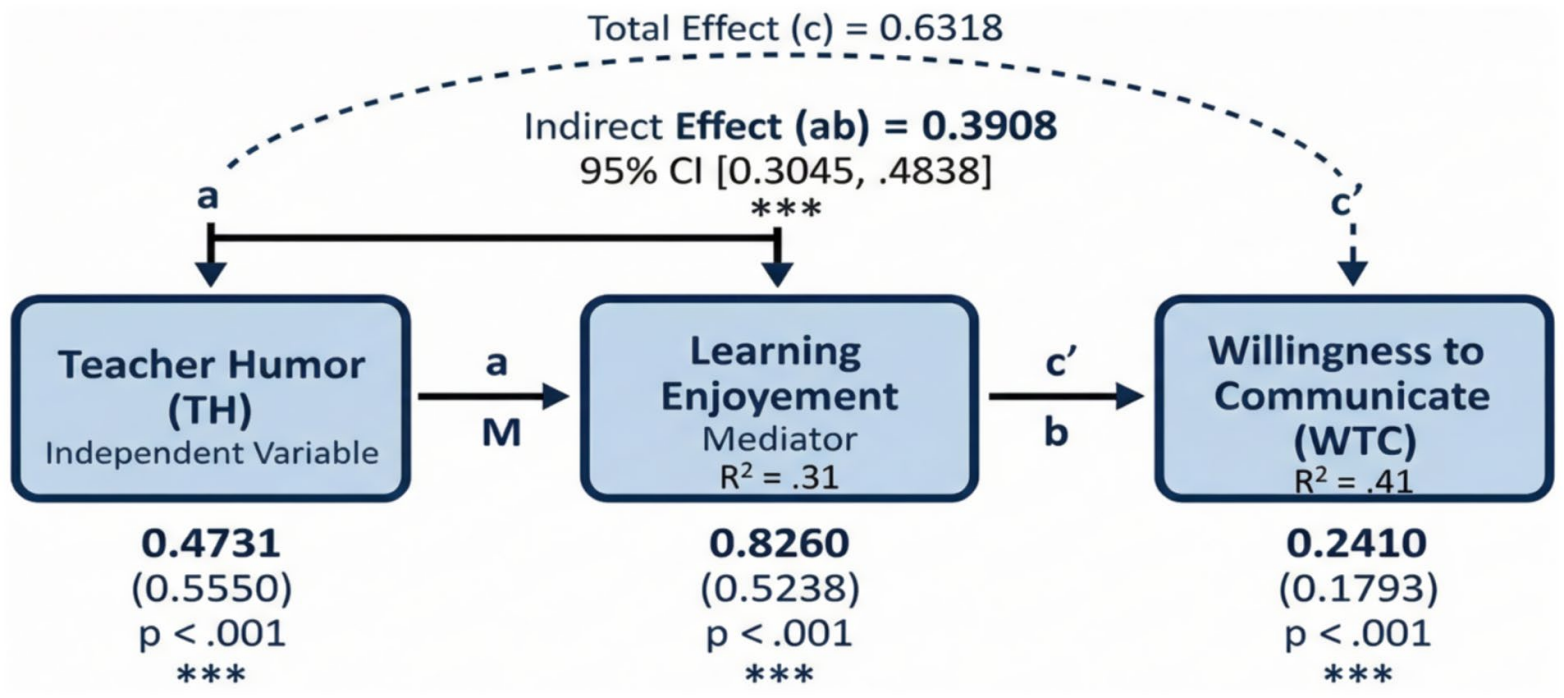
Verified mediation model of TH on WTC via ELE. Note: Unstandardized coefficients are shown outside parentheses, and standardized coefficients are presented in parentheses. R^²^ indicates the proportion of variance explained. ***p < 0.001. CI, Bias-Corrected Bootstrap Confidence Interval.

The results, presented in Table 10 and visually in Figure 2, revealed a significant total effect of TH on WTC (unstandardized effect = 0.63, *SE* = 0.05, *t* = 11.68, *p* < 0.001, 95% CI [0.53, 0.74]). As for the proposed mediation model: First, TH significantly predicted ELE, with an unstandardized coefficient (*B*) of 0.47 (*SE* = 0.03, *t* = 14.63, *p* < 0.001, 95% CI [0.41, 0.54]). This confirms the ‘a’ path of the mediation model, explaining 30.8% of the variance in ELE **(***R^2^* = 0.308). Second, when both TH and ELE were included in the model predicting WTC, ELE significantly predicted WTC (*B* = 0.83, *SE* = 0.07, *t* = 12.44, *p* < 0.001, 95% CI [0.70, 0.96]). This confirms the ‘b’ path.

**Table 10:** Mediation analysis results for the effect of teacher humor on willingness to communicate through English learning enjoyment.

Predictor	Outcome variable: ELE		Outcome variable: WTC	
	Unstandardized B (SE)	95% CI	Unstandardized B (SE)	95% CI
Constant	1.63 (0.13)***	[1.37, 1.89]	−0.81 (0.22)***	[−1.25, −0.38]
TH	0.47 (0.03)***	[0.41, 0.54]	0.24 (0.06)***	[0.13, 0.35]
ELE	–	–	0.83 (0.07)***	[0.70, 0.96]
*Effect analysis*	*Effect (SE)*	*t*	*95% CI*	
Total effect of TH on WTC	0.63 (0.05)	11.68***	[0.53, 0.74]	
Direct effect of TH on WTC	0.24 (0.06)	4.26***	[0.13, 0.35]	
Indirect Effect of TH on WTC through ELE	0.39 (Boot*SE* = 0.05)	–	[0.30, 0.48]	

*N* = 483. TH, teacher humor; ELE, English learning enjoyment; WTC, willingness to communicate. Coefficients reported are unstandardized. Bootstrap sample size = 5,000. CI, Confidence interval. ***p < 0.001.

Third, the indirect effect of TH on WTC through ELE was statistically significant (Effect = 0.39, Boot*SE* = 0.05, 95% BootCI [0.30, 0.48]). This result fully supports Hypothesis 3, indicating that ELE significantly mediates the relationship between TH and WTC. Finally, the direct effect of TH on WTC, while reduced, remained statistically significant (Effect = 0.24, *SE* = 0.06, *t* = 4.26, *p* < 0.001, 95% CI [0.13, 0.35]). This supports Hypothesis 4, suggesting that ELE partially mediates the relationship between TH and WTC. The total variance explained by the model predicting WTC was 41.08% (*R^2^* = 0.411).

Furthermore, regarding the potential confounding effects of demographic variables on the proposed mediation model, we conducted supplementary hierarchical regression analyses by including Gender and CET Level (as a proxy for objective English proficiency) as covariates. This step assesses the robustness of the core relationships (Humor Perception—ELE—WTC) when controlling for these factors.

The results of the preliminary regression steps showed that neither Gender (*B* range: −0.009 to 0.019, all *p* > 0.650) nor CET Level (*B* range: −0.051 to 0.060, all *p* > 0.130) were significant predictors of either English learning enjoyment (ELE) or Willingness to Communicate (WTC) across all models. This suggests that these variables do not act as major confounders in our data set.

Crucially, as shown in Table 11, the inclusion of Gender and CET Level did not substantially alter the significance or magnitude of the core paths in the mediation model:

1. Path a (predicting ELE): Teacher Humor (ETHPS_Affective) remained a highly significant predictor of ELE (*β* = 0.61, *p* < 0.001).
2. Path b (predicting WTC): When controlling for the covariates and the mediator (ELE), the direct effect of ELE on WTC remained highly significant (*β* = 0.83, *p* < 0.001, based on the main mediation model).

**Table 11:** Hierarchical regression analysis controlling for gender and CET Level.

Dependent variable	Predictor	*B* (SE)	*β*	*t*	*p*	*R^2^*	△*R^2^*
ELE	Step 1: covariates					0.003	0.003
	Gender	−0.009 (0.039)	−0.011	−0.269	0.788		
	CET level	0.023 (0.018)	0.06	1.515	0.13		
	Step 2: core Predictors					0.302	0.299***
	Teacher humor (Factor 1)	0.315 (0.052)	0.605	6.102	**<0.001**		
	(Factor 2)	0.147 (0.045)	0.203	3.268	**0.001**		
WTC	Step 1: covariates					0.004	0.004
	Gender	0.019 (0.056)	0.004	0.342	0.733		
	CET level	−0.004 (0.026)	−0.007	−0.165	0.869		
	Step 2: core predictors					0.211	0.207***
	Teacher humor (Factor 1)	0.392 (0.087)	0.298	4.533	**<0.001**		
	(Factor 2)	0.213 (0.075)	0.186	2.822	**0.005**		
	ELE (mediator)	0.83 (0.07)	–	11.68	**<0.001**		

*N* = 483. Gender: Reference category = [male/female]. The last row (ELE) is derived from the final step of the primary mediation model, demonstrating the robust prediction of WTC by the mediator after controlling for the covariates. Bold values in the *p* column indicate statistical significance at the *p* < 0.05 level. ***p < 0.001.

These findings confirm that the key direct and indirect relationships underpinning our mediation model are highly robust and are not confounded by the included demographic and objective ability measures. We can conclude that the significant indirect effect of Teacher Humor on WTC via ELE holds even when controlling for Gender and CET Level.

## 5 Discussion

### 5.1 The paradox of high enjoyment and low communication

The first research question investigated the current levels of ELE and WTC among Chinese college students. Our findings reveal a paradoxical pattern: moderately high ELE (*M* = 3.54) coexists with relatively low WTC (*M* = 3.08), suggesting a “high-affect-low-engagement” phenomenon among Chinese university EFL learners. This dissonance—wherein students derive pleasure from learning yet remain reluctant to engage in authentic communication—demands interpretation beyond simple correlation.

Our ELE finding aligns closely with Li and Han’s (2022a,b) report of comparable enjoyment levels (*M* = 3.59) across online and traditional classrooms, suggesting environmental invariance in positive affect. However, Li’s (2020) cross-cultural comparison positions Chinese learners’ enjoyment significantly below international averages, indicating that while our participants experience moderate enjoyment, systemic factors may constrain emotional expression in this context. The WTC result corroborates a persistent trend documented over 15 years: our data (*M* = 3.08) mirrors Wang’s (2015) “moderate” descriptor and Li and Zheng’s (2009) earlier finding (*M* = 2.82), confirming that communicative reluctance remains largely unchanged despite pedagogical innovations.

This affective-behavioral disconnect cannot be attributed solely to enjoyment deficits. Instead, it reflects what we term “affective surplus without behavioral yield”—a phenomenon where positive emotions fail to translate into communicative action. From a CVT perspective, this suggests that while humor may successfully enhance learners’ emotional experiences (raising perceived value), it may not sufficiently address control appraisals related to communication. Chinese learners may enjoy English lessons yet perceive low communicative competence or fear negative evaluation, which CVT identifies as separate pathways to behavioral outcomes (Pekrun, 2006).

Alternative explanations warrant consideration. Confucian values emphasizing other-directedness (tazhu, 他主) and face preservation structurally disincentivize spontaneous discourse (Wen and Clément, 2003). As Crozet and Liddicoat (1999) observed, communicative classrooms may paradoxically heighten anxiety when cultural congruence is lacking. Moreover, Chinese educational contexts often privilege high-stakes examinations over in-class participation (Peng, 2007), diminishing behavioral reinforcement for WTC. The disconnect may also reflect instructional emphases: if humor fosters enjoyment but lessons remain exam-focused rather than communicatively oriented, enjoyment may not channel into communicative courage. This interpretation is consistent with recent findings that classroom environment shapes WTC through multiple emotional pathways, with enjoyment being only one mediator alongside anxiety and boredom (Li et al., 2025).

Interestingly, students’ perception of teacher humor was notably high (*M* = 4.04), suggesting positive attitudes toward humor integration in English instruction. This finding supports earlier research demonstrating that appropriate humor strengthens teacher–student rapport (Tunnisa et al., 2019; Pranoto and Suprayogi, 2020) and aligns with recent evidence that humor strategies—particularly spontaneous humor and visual humor—are strongly endorsed when students hold positive attitudes toward in-class humor (Neff and Dewaele, 2023). However, high humor perception alone did not eliminate the WTC deficit, suggesting that rapport-building, while necessary, may be insufficient without targeted communicative scaffolding.

### 5.2 Teacher humor as a predictor of foreign language enjoyment

The second research question examined whether teacher humor significantly predicts students’ ELE. Results revealed a substantial positive relationship, consistent with prior research identifying teacher behaviors as significant influences on learners’ affective experiences (Dewaele and Dewaele, 2018; Li, 2022; Purwanti et al., 2024). More specifically, our findings empirically support qualitative observations that humor fosters emotional engagement and enhances learning pleasure (Fata et al., 2018; Farahani and Abdollahi, 2018). Recent experimental evidence demonstrates that humor reduces cognitive load and affective filtering (Peng, 2025), while studies in Indonesian contexts confirm humor’s role in enhancing motivation and engagement across listening comprehension tasks (Damanik et al., 2025).

Within the Control-Value Theory framework, humor functions as an external antecedent that reshapes learners’ appraisals along two dimensions. First, by reducing anxiety and making tasks more approachable, humor strengthens students’ perceived control over learning activities. Second, by highlighting meaningfulness and relevance—particularly when humor is curriculum-aligned—humor heightens perceived value of language learning (Bieg et al., 2017, 2019; Daumiller et al., 2020). These shifts in appraisal foster enjoyment, which CVT identifies as a core achievement emotion with downstream effects on motivation and behavior.

However, the humor-enjoyment relationship is not uniformly positive across all implementations. As Bao et al. (2023) cautioned, only content-integrated humor produces measurable engagement gains, while off-topic or self-disparaging jokes can nullify benefits. This aligns with Bieg et al.’s (2019) distinction between curriculum-relevant humor (which raises enjoyment) and aggressive humor (which reverses positive effects). Moreover, cultural variations in humor perception (Peng, 2007; Moalla, 2014) suggest that effectiveness depends on contextual appropriateness. Recent cross-cultural research reveals that humor strategies differentially affect learners depending on their foreign language enjoyment levels and attitudes toward classroom humor, with proficiency having minimal bearing (Neff and Dewaele, 2023). Additionally, Dewaele et al.’s (2022) longitudinal evidence indicates that prolonged humor absence leads to cumulative FLE declines, suggesting that consistency matters alongside appropriateness.

Individual differences may also moderate the humor-enjoyment link. Students with higher trait anxiety or lower initial enjoyment levels may respond differently to humor than more confident learners. Furthermore, the effectiveness of humor likely depends on teacher delivery skills and authenticity—forced or poorly timed humor may produce discomfort rather than enjoyment. Our aggregate finding, while statistically robust, obscures such heterogeneity and warrants future investigation of moderating variables.

### 5.3 The mediating role of enjoyment in the humor-WTC relationship

The third research question examined ELE’s mediating function between teacher humor and WTC. Results revealed a significant total effect of humor on WTC (*β* = 0.63, *p* < 0.001), confirming that teacher humor promotes communicative willingness, consistent with prior findings (Klassen et al., 2012; Thuy and Thao, 2022). More critically, this study identified ELE as a partial mediator through which humor influences WTC—humor first enhances ELE, which then predicts WTC. The mediation model demonstrated substantial explanatory power (*R^2^* = 0.41), accounting for 41% of WTC variance with a large effect size (Cohen, 1988).

This finding extends CVT in three theoretically meaningful ways. First, it positions teacher humor as a social-affective antecedent that shapes learners’ control-value appraisals, complementing CVT’s typical focus on task characteristics and self-perceptions (Pekrun and Raymond, 2014). Second, it demonstrates an affect-to-behavior conversion pathway: CVT emphasizes that achievement emotions link appraisals to outcomes (Pekrun, 2006), but applications have predominantly focused on cognitive performance. Our findings show that enjoyment also translates into social-interactional outcomes (Li et al., 2021), specifically communicative readiness in L2 classrooms. This addresses Li et al.’s (2025) recent observation that enjoyment serves as the strongest emotional mediator between classroom environment and WTC, surpassing anxiety and boredom. Third, by situating CVT within the L2 communicative context, our study demonstrates the theory’s relevance for understanding emotional underpinnings of participation—not merely achievement.

The exceptionally strong path from ELE to WTC (*β* = 0.83, *p* < 0.001) confirms earlier findings on the enjoyment-communication nexus (Zhang et al., 2024; Lin and Wang, 2025) and suggests that once enjoyment is established, communicative willingness follows robustly. This large effect aligns with Broaden-and-Build theory, which posits that positive emotions expand cognitive and behavioral repertoires (Fredrickson, 2001). The moderate path from humor to ELE (*β* = 0.47) indicates that while humor substantially influences enjoyment, other factors—such as teacher support, task design, and peer dynamics—also contribute. The additional direct path from humor to WTC, though smaller, suggests that humor may cultivate a classroom atmosphere conducive to communication independent of enjoyment (Klassen et al., 2012; Thuy and Thao, 2022). These positions humor as both a distal antecedent of appraisals and a proximal shaper of interactional readiness, enriching CVT by acknowledging socially dynamic learning environments.

Several caveats merit attention. First, partial mediation indicates that enjoyment does not fully explain humor’s effect on WTC. Unmeasured variables—such as communication confidence, anxiety reduction, or perceived teacher support—may constitute additional pathways. Zhang et al.’s (2024) research demonstrated that communication confidence and motivation mediate the FLE-WTC relationship, suggesting our model may benefit from incorporating these constructs. Second, the cross-sectional design precludes causal claims. While our model proposes that humor → enjoyment → WTC, alternative sequences are plausible: students with higher WTC may enjoy classes more, prompting teachers to use more humor (reverse causation). Solhi et al.’s (2024) experience sampling study revealed reciprocal relationships between teacher enjoyment and student FLE over time, cautioning against unidirectional assumptions. Third, our use of an undifferentiated humor measure constitutes a key limitation of the current study, as it does not distinguish between prosocial/affiliative and instructional/content-relevant styles identified in recent literature (Neff and Dewaele, 2023; Kabooha et al., 2024). Future research should examine whether these styles operate through distinct pathways or differentially predict WTC.

### 5.4 Theoretical and practical contributions

This study contributes to theory and practice in several ways. Theoretically, it extends CVT by demonstrating that teacher humor—a social-affective factor—functions as an external antecedent shaping learners’ control-value appraisal. It advances CVT’s application by establishing an affect-to-behavior conversion pathway, showing that enjoyment predicts not only cognitive outcomes but also communicative readiness. It situates CVT within L2 communicative contexts, broadening the theory’s relevance beyond achievement-focused settings.

Pedagogically, findings suggest that humor is not merely entertaining but a theoretically grounded tool for enhancing emotional experiences and stimulating participation. However, effectiveness depends on implementation quality. As recent research emphasizes, humor should be curriculum-relevant (Bao et al., 2023), culturally appropriate (Peng, 2007; Moalla, 2014), and strategically deployed to foster both enjoyment and communicative confidence (Zhang et al., 2024). Given the persistent affective-behavioral gap in Chinese EFL contexts, instructors should implement “affective bridging”—designing tasks that strategically channel enjoyment into communicative courage rather than assuming enjoyment automatically yields participation (Xia and Zhou, 2020). This might involve pairing humor with low-stakes communicative activities, scaffolding speaking tasks to enhance control appraisals, and providing positive feedback that reinforces both enjoyment and efficacy.

Our findings emerge from Chinese university contexts where Confucian values and exam-oriented education shape learning behaviors. While FLE strongly predicts WTC among Saudi students, grit exerts greater influence among Moroccan students (Bensalem et al., 2025), suggesting that affective interventions like humor may operate differently across cultural contexts. Educators must therefore adapt humor strategies to local norms, student populations, and institutional priorities rather than applying universal prescriptions.

## 6 Conclusion and recommendation

This study investigated relationships among teacher humor, foreign language enjoyment, and willingness to communicate among Chinese university EFL learners. Three principal findings emerged: (1) students exhibited moderate ELE (*M* = 3.54) and high perceived teacher humor (*M* = 4.04), yet WTC remained relatively low (*M* = 3.08), revealing an affective-behavioral gap; (2) teacher humor strongly predicted ELE (*β* =0.548, *p* <0.001); and (3) ELE partially mediated the humor-WTC relationship (*R^2^* =0.41), demonstrating that humor enhances communicative willingness primarily through fostering enjoyment.

These findings extend Control-Value Theory into L2 communicative contexts by establishing an empirically validated pathway linking teacher social-affective behaviors to student emotional and behavioral outcomes. Pedagogically, results suggest that humor constitutes a theoretically grounded tool for enhancing classroom emotional climate and participation. However, effectiveness depends on strategic implementation: humor should be curriculum-relevant, culturally appropriate, and paired with communicative scaffolding to translate enjoyment into speaking behavior.

Despite these findings, several limitations must be acknowledged. First, concerning the scope of the study, our investigation was primarily limited to examining the relationship between English teacher humor and university students’ learning enjoyment. This scope does not allow for the exploration of important contextual and qualitative nuances, such as potential side effects, cultural considerations (Jiang, 2022), or the specific types of humor used (Bieg et al., 2017, 2019) that may prevent inappropriate humor from diminishing students’ learning enjoyment. Future studies should broaden this focus by examining how humor interacts with other emotional and motivational constructs, testing different humor styles, and investigating cross-cultural generalizability.

Second, two methodological constraints require careful interpretation of the findings. The reliance exclusively on self-report questionnaires administered in a single survey session raises the potential for Common Method Variance (CMV). While this risk of inflated correlations and social-desirability bias exists, we statistically assessed this threat using Harman’s single-factor test. Results indicated that the first unrotated factor accounted for 43.630% of the total variance, which is below the conservative 50% threshold, suggesting that CMV is not a severe threat to the validity of our findings. Nevertheless, to fully address this limitation, future research should employ multi-method designs, incorporating measures such as teacher-rated humor, observer-rated Willingness to Communicate (WTC), or behavioral indicators of communication to provide a more objective assessment.

Furthermore, the cross-sectional design restricts our ability to draw causal inferences. While the mediation model provides a theoretical framework for understanding these associations, the directionality cannot be empirically verified with the current data. Future research utilizing longitudinal designs or experimental methods is recommended to confirm the proposed pathways suggested by our findings.

## Data Availability

The raw data supporting the conclusions of this article will be made available by the authors, without undue reservation.

## Footnotes

1
https://www.wjx.cn
