# Supplementary material for: A perplexing silence: unlocking student communication through teacher humor in the EFL classroom
Source: Front Psychol. 2026 Jan 12;16:1721941. doi: 10.3389/fpsyg.2025.1721941 (PMC12832323; doi:10.3389/fpsyg.2025.1721941)
Supplement: Supplementary file 1 [file Data_Sheet_1.docx]

**A****ppendix 1:** **﻿The English Teachers’ Humor Perception Scale (ETHPS) (Student form)**

| **No.** | **﻿因子和题项** | ﻿**Factors and Items** | ﻿**Factor loading** |
| --- | --- | --- | --- |
|  | **因子1: 情感互动效能**  **Factor 1: Affective Interaction** **Efficacy** | |  |
| 1 | 英语教师幽默拉近了师生心理距离。 | The English teacher’s humor effectively bridged the psychological gap between teachers and students. | 0.857 |
| 2 | 英语教师幽默让课堂氛围更轻松愉悦。 | The English teacher’s humor created a more relaxed and enjoyable classroom atmosphere. | 0.844 |
| 3 | 英语教师幽默增强了我的课堂参与意愿。 | The English teacher’s use of humor significantly boosted my willingness to engage in class. | 0.824 |
| 4 | 英语教师的幽默帮助我更好记住知识点。 | The English teacher’s humor improved my retention of key knowledge points. | 0.820 |
| 5 | 英语教师常通过幽默鼓励内向的同学参与课堂。 | The English teacher often uses humor to encourage shy students to participate. | 0.743 |
| 6 | 英语教师会根据学生的水平灵活调整幽默风格。 | The English teacher adapts humor to suit different student proficiency levels. | 0.734 |
| 7 | 英语教师的幽默帮助我减轻用英语发言的焦虑。 | The English teacher’s humor helps reduce my anxiety about speaking English. | 0.727 |
| 8 | 英语教师在幽默形式上多样化（故事、视觉辅助、模仿等）。 | The English teacher varies the types of humor (stories, visual aids, parody). | 0.702 |
| 9 | 当英语教师使用幽默时，我感觉与同学之间的关系更融洽。 | When the English teacher uses humor, I feel more connected to classmates. | 0.686 |
|  | **因子2:** **幽默实施效能**  **Factor 2: Humor Implementation Efficacy** | |  |
| 10 | 我觉得英语教师的笑话非常有趣。 | I find my English teacher’s jokes genuinely funny. | 0.819 |
| 11 | 我的英语教师通过角色扮演、游戏等有趣活动让我们发笑。 | My English teacher employs playful activities (e.g. role-plays, games) to make us laugh. | 0.819 |
| 12 | 我的英语教师定期在课堂上讲笑话或有趣的故事。 | My English teacher tells jokes or funny stories during class regularly. | 0.808 |
| 13 | 我的英语教师在讲解新语法点时经常使用幽默的例子。 | My English teacher uses humorous examples when explaining new grammar points. | 0.795 |
| 14 | 英语教师的幽默让我期待每一次上课。 | The English teacher’s humor turned each class into an experience I eagerly anticipated. | 0.712 |
| 15 | 英语教师的幽默提升了我记忆单词和句型的能力。 | The English teacher’s humor enhances my ability to remember words and sentence patterns | 0.691 |

**﻿**Note: The Sense of English Teacher’s Humor Scale (SETHS) is a 5-point Likert scale ranging from 1 “strongly disagree” to 5 “strongly agree”.
